# Immunogenicity of a Candidate Hepatitis C Vaccine Based on Non-Structural DNA-Protein Sequences and a Novel Complex Adjuvant

**DOI:** 10.3390/vaccines14070640

**Published:** 2026-07-21

**Authors:** Olga V. Masalova, Ekaterina I. Lesnova, Vyacheslav V. Kozlov, Vladimir T. Valuev-Elliston, Kristina Yu. Permyakova, Natalya E. Fedorova, Tatyana N. Nikolaeva, Alexander V. Pronin, Alexander V. Ivanov, Alla A. Kushch

**Affiliations:** 1 Gamaleya National Research Center of Epidemiology and Microbiology, Ministry of Health of the Russian Federation, 123098 Moscow, Russia; 2 Engelhardt Institute of Molecular Biology, Russian Academy of Sciences, 119991 Moscow, Russia; 3 Federal State Budgetary Educational Institution of Higher Education “Moscow State Academy of Veterinary Medicine and Biotechnology—MVA by K.I. Skryabin”, 109472 Moscow, Russia

**Keywords:** hepatitis C virus (HCV), nonstructural HCV proteins, immune response, HCV vaccine, DNA immunization, adjuvants, memory T cells, myeloid derived suppressor cells (MDSCs), regulatory T cells (Tregs)

## Abstract

Global elimination of hepatitis C virus (HCV) infection requires not only direct-acting antivirals (DAAs) but also the development of a highly effective prophylactic and/or therapeutic vaccine. **Background/Objectives**: Our aim was to optimize the composition of the candidate vaccine against HCV by combining recombinant non-structural proteins and a DNA construct with a complex adjuvant. **Methods**: C57BL/6 and DBA/2J mice were immunized three times at 2-week intervals using different schemes. The viral antigens consisted of a mixture of NS3, NS5A, and NS5B proteins and/or recombinant DNA expressing NS3-NS5B polyprotein. As adjuvants, a complex adjuvant, a mixture of Polymuramil^®^ and Pyrogenalum^®^ (NOD1/NOD2 and TLR-4 agonists), or a CpG ODN adjuvant (TLR-9 agonist) were used. **Results**: The most efficient regimen was three subcutaneous administrations of the combined DNA, recombinant protein components, and a new complex adjuvant. This scheme elicited a robust immune response, characterized by high antibody titers, enhanced antigen-specific lymphocyte proliferation, and significant interferon-gamma (IFN-γ) secretion in both mouse lines. Furthermore, the complex adjuvant outperformed CpG ODN in stimulating both humoral and cellular immunity against the HCV antigens. The vaccine composition stimulated the formation of CD4^+^ memory T cells and decreased the relative frequences of suppressive Treg and MDSCs. **Conclusions**: The presented candidate vaccine induces a strong immune response to HCV proteins. The next step would be to validate the protective effect in cell culture and animal models. This would one the path to preclinical studies of this vaccine composition.

## 1. Introduction

Hepatitis C caused by the hepatitis C virus (HCV) is one of the major causes of chronic liver disease, including end-stage liver disease—cirrhosis and hepatocellular carcinoma [1,2,3]. Approximately 80% of cases of acute infection develop into a chronic disease. Despite the breakthrough in the treatment of chronic hepatitis C (CHC) with direct-acting antivirals (DAAs) that clear the infection in more than 95% of patients [4,5,6,7,8,9,10,11], the global incidence of new infections and reinfections remains high. This is attributed to the fact that viral clearance via DAAs does not confer protective immunity against subsequent exposure [1]. In addition, there are well-documented cases of occult infection [12], and DAAs do not prevent subsequent reactivation of the virus from reservoirs. Even in patients who achieve SVR, there is an increased rate of further liver disease progression [13]. The lack of a prophylactic vaccine is considered the main reason for ineffective hepatitis C control.

The development of a hepatitis C vaccine is hampered by several factors, such as high genomic diversity, the emergence of quasispecies harboring mutations in T- and B-cell epitopes during immune pressure, and limitations of cell culture models of the virus. Furthermore, the field is constrained by a lack of immunocompetent animal models to test the effectiveness of candidate vaccines and by ambiguity in correlates of immune protection [14,15]. An ideal vaccine should elicit a potent, multi-epitope, functional cellular immune response and virus-neutralizing antibodies. To date, however, no candidate vaccine has demonstrated sufficient efficacy in preventing chronic infection [14,16].

Currently, there is no consensus regarding the optimal antigenic composition for an HCV vaccine. However, non-structural (NS) proteins 3, 4A, 4B, 5A, and 5B are essential for viral replication and are highly immunogenic. These proteins are therefore promising targets for a cellular immune response. Unlike highly variable E1 and E2 envelope glycoproteins, NS proteins harbor highly conserved regions with CD4^+^ and CD8^+^ T-cell epitopes [17,18]. Therefore, such a vaccine should induce not the neutralizing antibodies but the robust cell immunity.

There are various approaches to the development of vaccines against HCV [15]. While inactivated or attenuated whole-virus vaccines are under investigation, their development is limited by cell culture models restricted to specific JFH1-based recombinant subtypes (genotype 2a) [19]. Infectious clones of genotype 1b (e.g., GLT1cc, Con1cc)—the most prevalent and clinically significant genotype in Russia and several other regions—remain largely inaccessible for large-scale propagation [20,21]. Therefore, the development of vaccines relies on genetically engineered constructs that ensure the expression of HCV proteins, recombinant proteins, and DNA-based viral vectors [15,22,23].

While proteins predominantly induce humoral (Th2) immunity, plasmids favor cellular (Th1) immunity. Due to their modest immunogenicity, both platforms require the use of adjuvants [15,22]. Multiple lines of evidence suggest that heterologous prime-boost or combined DNA-protein regimens induce a more robust and balanced immune response compared to monotypic formulations [24,25]. This synergy likely results from a broader engagement of pathogen-associated molecular patterns (PAMPs) with host pattern recognition receptors (PRRs), including membrane-bound Toll-like receptors (TLRs) and cytosolic nucleotide-binding oligomerization domain (NOD)-like receptors (NLRs) containing either protein 1 (NOD1) or protein 2 (NOD2). The activation of these PRR signaling pathways triggers the production of pro-inflammatory cytokines essential for priming adaptive immunity [26].

Previously, we demonstrated that immunization of mice with recombinant NS3 and NS5B proteins and the pcNS3-NS5B plasmid encoding the respective polyprotein of the virus enhanced both cellular and humoral responses [27]. In those studies, N-acetylglucosaminyl-N-acetylmuramyl-L-alanyl-D-isoglutamine (GMDP, NOD2 receptor) or IFN-α were used as adjuvants for the proteins, while the DNA construct was combined with a plasmid encoding granulocyte-macrophage colony-stimulating factor (GM-CSF). Later, we reported that a complex adjuvant targeting both TLR4 and NOD1/NOD2 synergistically enhances the immunogenicity of individual NS proteins [28]. TLR4 interacts with bacterial lipopolysaccharides (LPS) to drive potent immune activation. A pharmaceutical-grade TLR4 ligand used in Russia is Pyrogenalum^®^ (PG)—an LPS from *Salmonella typhi*. Concurrently, NLRs recognize bacterial peptidoglycans and viral RNA [29]. A novel peptidoglycan-based product, Polymuramil^®^ (PM), interacts with both NOD1 and NOD2, offering a distinct advantage over single-receptor agonists [30]. All this warrants a comparison of these adjuvant systems—specifically the aforementioned complex adjuvant—to evaluate their efficacy in modulating the immune response to a combined DNA and recombinant non-structural protein vaccine.

The aim of the work was to enhance the immunogenicity of a candidate HCV vaccine based on non-structural sequences by optimizing the formulation composition and the route of administration.

## 2. Materials and Methods

### 2.1. Animals

Mice of the C57BL/6 (H-2b) and DBA/2J (H-2d) lines (females, 6–8 weeks old) were purchased from the Stolbovaya breeding and nursery laboratory (Research Center for Biomedical Technologies of FMBA, Stolbovaya, Moscow Region). All animal experiments were carried out in accordance with Order No. 199n, “Introduction of good laboratory practice” of the Ministry of Health of the Russian Federation, with the “Regulations on the Biomedical Ethics Committee of the N.F. Gamaleya National Center for Epidemiology and Microbiology (Russia)” (approval code No. 11-22-05-2025), and Directive 2010/63/EU of the European Parliament and of the Council.

### 2.2. The Recombinant HCV Proteins

The recombinant NS4 (1677–1754 aa) and NS5A (2061–2302 aa) HCV proteins of genotype 1b were purchased from the R&D company “Diagnostic systems” (Nizhniy Novgorod, Russia). The recombinant NS3 protein (helicase domain 1230–1658 aa, genotype 1b); and the NS5B protein lacking the C-terminal hydrophobic 21 amino acid residues (2420–2990 aa, genotype 1b) were expressed in *Escherichia coli* and purified on Ni-NTA agarose as described previously [31,32]. Protein concentrations were measured using the Bradford assay (Sigma, Darmstadt, Germany). The recombinant HCV proteins were used as virus-specific components for mouse immunization, as antigens to stimulate T-cell responses in vitro, and as sorbents in an enzyme-linked immunosorbent assay (ELISA) to evaluate antibody production.

### 2.3. Synthetic Peptides

The peptides representing known HCV CTL epitopes were combined into four pools: NS3 (aa 1104–1123, 1203–1222, 1363–1454, 1447–1466), NS4 (aa 1689–1712, 1693–1707, 1764–1772, 1788–1798, 1921–1940), NS5A (aa 2140–2149, 2151–2160, 2163–2171, 2225–2233, 2252–2260, 2269–2277, 2295–2317), and NS5B (aa 2555–2563, 2576–2584, 2594–2601, 2726–2733, 2820–2827). The peptides were synthesized at BelkiAntitela.RF LLC (Stavropol, Russia) and at the Institute of Bioorganic Chemistry of the Russian Academy of Sciences, and were used to stimulate the cellular response in vitro.

### 2.4. Plasmid

We used the pcNS3-NS5B plasmid construct encoding five non-structural HCV proteins (NS3, NS4A, NS4B, NS5A, and NS5B) of genotype 1b based on a commercial pcDNA-3.1(+) vector (Invitrogen, Carldbad, CA, USA) [33,34]. The plasmid was purified from the E. coli JM109 strain with the QIAGEN Plasmid Purification Maxi Kit (QIAGEN, Hilden, Germany) according to the manufacturer’s instructions.

#### The Compounds and Adjuvants

A combination of two drugs was used as a complex adjuvant: Polymuramyl^®^ (PM), NOD1/NOD2 ligand, which is a mixture of three active components from the peptidoglycan of the cell wall of Gram-negative bacteria *S. typhi* (Medgamal Branch of the Gamaleya National Research Center of Epidemiology and Microbiology of the Ministry of Health of the Russian Federation, 200 μg/0.5 mL, series 011-0219); Pyrogenal^®^ (PG)–LPS, TLR4 ligand, isolated from *S. typhi* (Combiotech Research and Production Company, Russia,100 μg/mL, series 164). The drugs approved for medical use were purchased from a local pharmacy. For comparison, a licensed adjuvant representing a synthetic oligonucleotide with unmethylated CpG motifs (ODN 1826, CpG (InvivoGen, San-Diego, CA, USA)) was used as a TLR9 agonist.

### 2.5. Mouse Immunization

Recombinant HCV proteins (NS3, NS5A, and NS5B) were injected subcutaneously in the tail root region (SC) or intramuscularly into the quadriceps femoris muscle (IM), each at a dose of 2 μg/mouse, in saline or in a mixture with adjuvants. The optimal ratios of compounds for a complex adjuvant were previously determined experimentally: the mixture consisted of PM—20 μg/mouse and PG—15 μg/mouse [28]; the dose of CpG is 50 μg/mouse [35]. The pcNS3-NS5B plasmid was administered at a dose of 100 μg/mouse SC or IM. The recombinant proteins with adjuvants and the plasmid were administered in a mixture or separately, as described in the Section 3. The control groups of mice were administered saline following the same schedule. Each group of mice consisted of 5–8 animals. Three immunizations with an interval of 2 weeks were conducted, and the immune response was assessed 7–9 days after the last injection.

### 2.6. Humoral Immune Response

The levels of specific antibodies in mouse serum samples were quantified by indirect ELISA as described previously [28,34,36]. Briefly, 96-well plates (MaxiSorp, Nunc, Roskilde, Denmark) were coated with the recombinant HCV proteins (1 µg/mL in PBS, pH 7.4) during overnight incubation at 20 °C. Serial dilutions of sera done in triplicate were incubated for 1 h at 37 °C. Anti-HCV antibodies were detected using antibodies against mouse Ig isotypes IgG1 and IgG2a conjugated with horseradish peroxidase (Jackson Immunoresearch Laboratories, West Grove, PA, USA). Sera and secondary antibodies were diluted in the blocking buffer (PBS containing 1% casein and 0.1% Tween-20). After each incubation, the plates were washed five times with PBS supplemented with 0.1% Tween-20. Antibody levels were assessed using 3,3′,5,5′-tetramethylenbensidine (TMB; BioTestSystems, Moscow, Russia). As the serum titer in ELISA, we used the reciprocal of the highest serum dilution, at which the optical density at 450 nm was two times higher than that of the control group.

### 2.7. T-Cell Proliferation and ELISpot Assays

Spleens of mice belonging to one group were homogenized, and pooled splenocytes were used to assess cellular responses. T-cell proliferation was quantified by measuring the incorporation of [^3^H]-labeled thymidine into cell DNA according to the previously published protocol [34,36]. As specific stimulants, we added four pools of the NS3, NS4, NS5A, and NS5B peptides and the respective recombinant viral proteins at a 1 µg/mL concentration. The results are expressed as ratios of mean [^3^H] incorporation in the presence of antigens to mean [^3^H] incorporation in wells with fresh medium used as a negative control.

Quantification of the cells producing IFN-γ was performed using a Mouse IFN-γ ELISpotPLUS kit (HRP) (Mabtech, Nacka Strand, Sweden), again as described previously [36].

### 2.8. Cytokine Quantification

Cytokine levels in cell culture supernatants obtained after the stimulation of lymphocytes were measured by ELISA using a High Sensitive ELISA Kit for Interferon Gamma (Cloud-clone, Wuhan, China) in accordance with the manufacturer’s instructions. It allowed the detection of IFN-γ with a sensitivity of 2 pg/mL. The concentrations of IFN-γ were calculated from the respective calibration curves obtained using the standards.

### 2.9. Flow Cytometry

The phenotype of cells from mouse spleens was determined using multicolor flow cytometry as described earlier [36] with few modifications. CD25 was detected using a fluorescently labeled antibody clone PC61 from BD Biosciences (Milpitas, CA, USA). To determine MDSC, a population of lymphocytes was isolated, and cells from this gate were used to identify CD11c(-) cells. Dot plot CD11c(-) was used to determine the proportion of Gr-1+, CD11b+ cells.

### 2.10. Statistical Analysis

All values were given as means ± SD of three independent experiments. Statistical analysis was carried out with Statistica 10 (StatSoft Inc., Tulsa, OK, USA). All graphs were designed using GraphPad Prism v.10 (Graphpad Software Inc., San Diego, CA, USA, version 8.0). The significance of differences was analyzed either by a two-tailed Student’s *t*-test or a Mann–Whitney test. The difference between multiple groups was analyzed by ANOVA with Tukey’s post hoc test.

## 3. Results

### 3.1. The Complex Adjuvant PM + PG Is Superior to CpG ODN in Terms of Effectiveness When Immunizing Mice with a Mixture of Non-Structural HCV Proteins

We previously demonstrated that the combined administration of Polymuramil (PM) and Pyrogenalum (PG) as a complex adjuvant exerts a synergistic effect when used with individual recombinant HCV non-structural proteins (NS3, NS4, or NS5B) [28]. Their combination increased antibody titers and enhanced antigen-specific lymphocyte proliferation and IFN-γ secretion in vitro. The effect of the complex adjuvant on the NS5A protein, as well as on the combination of non-structural HCV proteins, has not been studied so far. In this study, the immunostimulatory activity of the PM + PG complex was compared to a licensed adjuvant from the group of TLR9 agonists, CpG. C57BL/6 mice were immunized subcutaneously with a mixture of three HCV proteins (NS3, NS5A, and NS5B) combined with saline without adjuvants (group 1), with CpG (group 2), and with PM + PG (group 3). A control group (4) was injected with saline only.

**Figure 1:**
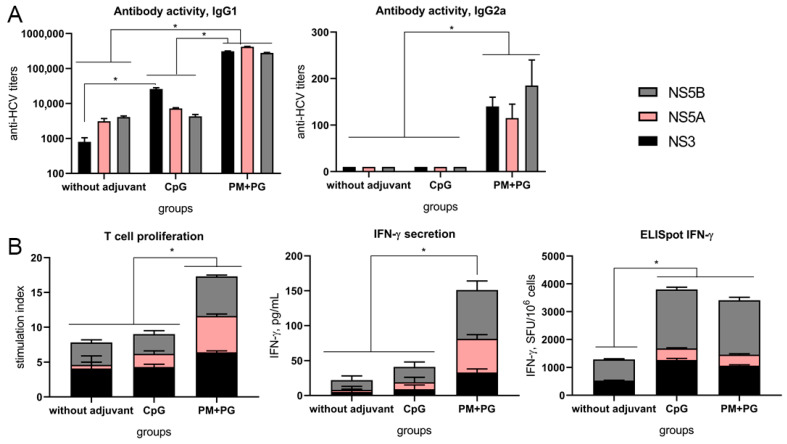
Comparison of the effectiveness of adjuvants when immunizing C57BL/6 mice with a mixture of non-structural HCV proteins. Each group (five mice) was subcutaneously injected with a mixture of three HCV proteins (NS3, NS5A, NS5B) in combination with saline without adjuvants, with CpG, with PM + PG, and a control group injected with saline only. (**A**)—humoral response, the levels of anti-HCV antibodies in the sera of mice are expressed as endpoint titers in ELISA; data presented as geometric mean titers of IgG1 and IgG2a antibody ± standard deviations (SD). (**B**)—Results of cellular response reactions of lymphocytes of immunized mice to stimulation with recombinant proteins in vitro were assayed as follows: proliferative activity was expressed as stimulation indexes (SI); secretion of IFN-γ in ELISA—in pg/mL; the number of IFN-γ-synthesizing cells by ELISpot in the number of spot-forming units (SFU) per 10^6^ cells. Data are presented as means ± SD from three independent experiments. * *p* < 0.05 between the specified groups (ANOVA with Tukey’s test).

Immunization with the recombinant HCV proteins in the absence of adjuvants elicited exclusively IgG1 antibodies, with titers ranging from 1:800 to 1:4 × 10^3^ (Figure 1A). The CpG adjuvant caused a 30-fold increase in NS3-specific IgG1 activity. The complex PM + PG adjuvant enhanced IgG1 titers across all three HCV proteins by several orders of magnitude. Furthermore, the PM + PG formulation also induced IgG2a production, albeit at lower titers. No HCV-specific antibodies were detected in the control group. Neutralizing activity of these antibodies was not evaluated, as virions lack non-structural proteins.

In mice immunized with HCV proteins without adjuvants (group 1), lymphocyte proliferation was observed only in response to stimulation with recombinant NS3 and NS5B proteins (Figure 1B). While CpG increased lymphocyte proliferation only in response to NS5A protein, the Stimulation Index (SI) did not change significantly. The PM + PG combination significantly increased lymphocyte proliferation in response to NS3, NS5A, and NS5B. IFN- γ secretion by stimulated lymphocytes in group 1 was observed only in response to NS5B (Figure 1B). The use of CpG did not significantly improve this response; however, the addition of PM + PG increased cytokine production for all three HCV proteins.

In the ELISpot assay, recombinant NS3 and NS5B proteins without adjuvants stimulated IFN-γ production in a subset of spleen cells from group 1 mice (Figure 1B). Both CpG and PM + PG adjuvants (groups 2 and 3) significantly increased the number of IFN-γ secreting cells in response to all recombinant proteins. Notably, ELISpot is recognized as the most informative method recommended for assessing the T-cell response to new hepatitis C vaccines [37].

Therefore, our data show for the first time that the proposed combination of Polymuramyl and Pyrogenalum compounds stimulates both humoral and cellular immune responses to three non-structural HCV proteins administered at a low dose (2 μg/mouse). Importantly, this complex adjuvant outperformed the well-known CpG ODN adjuvant in most parameters.

### 3.2. Simultaneous Subcutaneous Administration of Candidate Vaccine Components Elicits the Greatest Cellular Immune Response

To enhance the immune response, experiments were conducted to compare various administration schedules for the candidate vaccine components. The administration schedules are presented in Table 1. During these in vivo experiments, no mortality or weight loss among the immunized mice was registered. Furthermore, the behavioral responses of the experimental groups did not differ from those of the control groups, suggesting that the administered formulations are safe.

**Figure 2:**
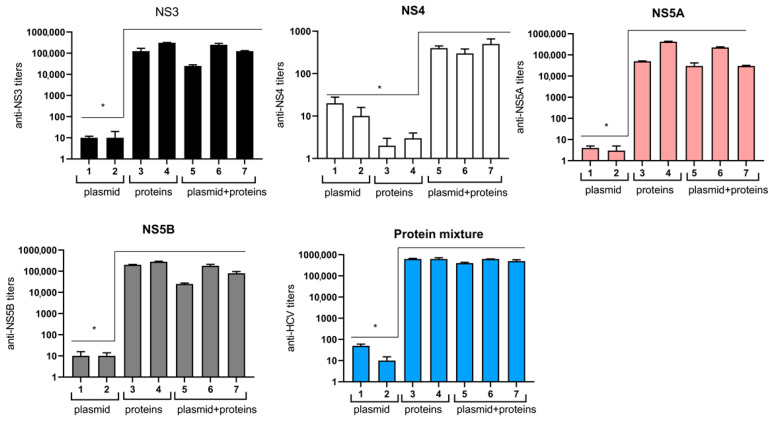
Induction of IgG1 antibodies to HCV proteins in C57BL/6 mice receiving different HCV immunogens using intramuscular or/and subcutaneous injections. C57BL/6 mice were immunized three times at 2-week intervals with either individual vaccine components (a mixture of recombinant NS3, NS5A, and NS5B HCV proteins or pcNS3-NS5B plasmid), or a combination of these components. The combinations were administered intramuscularly and/or subcutaneously. Four recombinant HCV proteins (described in Materials and Methods) or a protein mixture were used as sorbents in ELISA to evaluate antibody production (IgG1 antibody isotype). Values show the geometric mean titer ± SD of three measurements done from three independent experiments. * *p* < 0.05 between the specified groups (ANOVA with Tukey’s test).

The results demonstrate that active production of antibodies required the inclusion of the recombinant proteins in the immunization schemes (groups 3–7) (Figure 2). In these groups, IgG1 titers against NS3, NS5A, and NS5B reached 4 × 10^5^. The highest level of antibody activity was observed in groups 3 and 6, where the proteins were administered subcutaneously. In contrast, the plasmid induced a very weak humoral response (*p* < 0.05). Regarding antibodies to NS4, the combination of the plasmid and the proteins also induced antibody production (groups 5–7), with the total titer against non-structural HCV proteins exceeding 6 × 10^5^. Notably, the titers of IgG2a antibodies were significantly lower (≤150). In the control groups (8 and 9), no humoral response to HCV antigens was detected (≤10).

**Figure 3:**
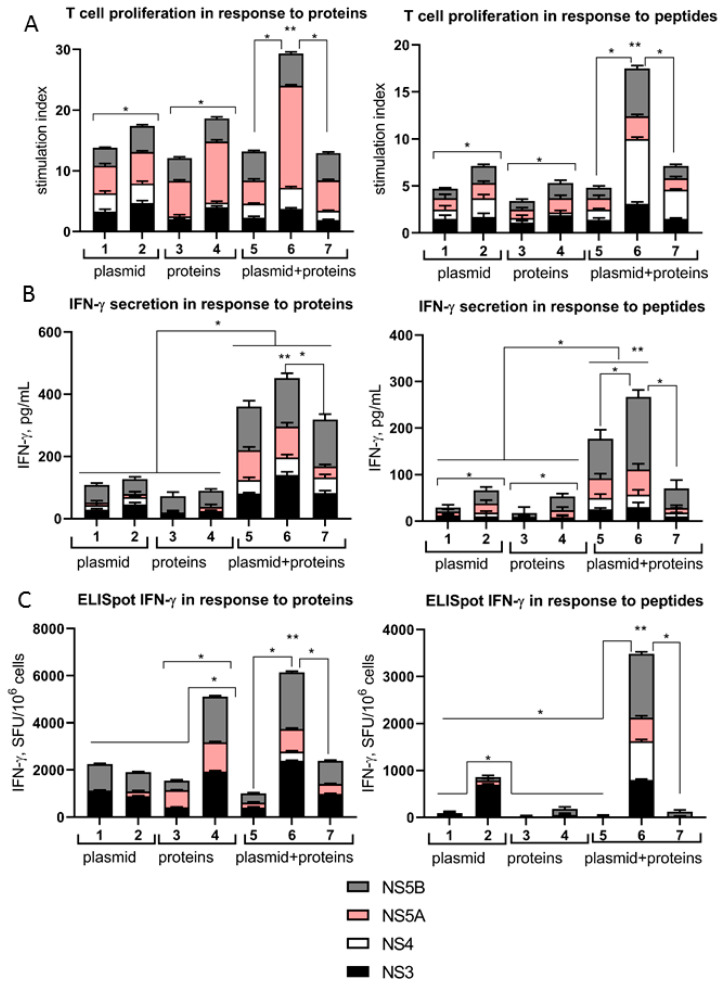
The cellular immune response in mice immunized with non-structural HCV proteins and/or plasmid pcNS3-NS5B with a complex adjuvant varies depending on the administration regimen of the candidate vaccine components. C57BL/6 mice were immunized three times at 2-week intervals with either individual vaccine components (a mixture of recombinant NS3, NS5A, and NS5B HCV proteins or pcNS3-NS5B plasmid) or a combination of these components. The preparations were administered intramuscularly and/or subcutaneously. Results of cellular response reactions of lymphocytes of immunized mice to stimulation with recombinant proteins and peptides in vitro: (**A**) proliferative activity is expressed as stimulation indices (SI); (**B**) secretion of IFN-γ in ELISA—in pg/mL; (**C**) the number of IFN-γ-synthesizing cells by ELISpot in the number of spot-forming units (SFU) per 10^6^ cells. Data are presented as means ± SD. * *p* < 0.05 between the specified groups (Student’s *t*-test and ANOVA with Tukey’s test); **—maximum values compared to the other groups (*p* < 0.05, ANOVA with Tukey’s test).

To assess the cellular immune response, the recombinant proteins and peptide mixtures representing CD8^+^ T-cell epitopes were used separately as stimulants. The lymphocytes from mice immunized with the plasmid (group 2) showed significant proliferation in response to stimulation with the recombinant proteins, with the SI in group 2 being significantly higher than the SI in group 1 (Figure 3A). A similar proliferative response was observed in groups 3 and 4, which were injected with recombinant proteins, as well as in groups 5 and 7. In contrast, in group 6, where the proteins and plasmid were injected together subcutaneously, the SIs were significantly higher than in the other groups of mice. In response to peptide stimulation, SIs were also higher in groups 2 and 4 compared to groups 1 and 3. In group 6, the SI increased by 2.5–3-fold in response to peptides from the sequences of all proteins studied.

Figure 3B illustrates the concentrations of IFN-γ secreted by lymphocytes into the culture medium following stimulation with the HCV antigens. When lymphocytes were stimulated with the peptides, the trend was similar to that of the proliferation assay: the cytokine signal in groups 2 and 4 was significantly higher than in groups 1 and 3, respectively. Notably, IFN- γ secretion in group 6 was the highest among all experimental groups. In the ELISpot assay, the recombinant proteins induced a high number of IFN-γ-producing cells in group 4, with even higher numbers registered in group 6 (Figure 3C). In response to peptide stimulation, spots were primarily detected in group 2 (with the highest frequency in the case of NS3), while the maximum number of spots was recorded in group 6 (for all protein sequences) after subcutaneous immunization with both proteins and plasmids. In the control groups (8 and 9), no cellular response to HCV antigens was detected.

Thus, triple subcutaneous immunization of animals with a mixture of plasmids, recombinant proteins, and a complex adjuvant represents the most effective vaccination scheme for the induction of both humoral and cellular immune responses.

### 3.3. The Candidate Vaccine Induces an Effective Immune Response in Genetically Diverse Mice

It is known that the direction of T-cell responses to viral and bacterial antigens differs in mice with different MHC antigens: BALB/c and DBA/2J (H-2d haplotypes) have a predominantly Th2-type immune response, while C57BL/6 (H-2b) has a predominantly Th1-type response [38,39]. The experiments described above were conducted in C57BL/6 mice. Therefore, the next step was to evaluate the immunogenicity of the optimized DNA-protein-adjuvant formulation in the DBA/2J strain.

**Figure 4:**
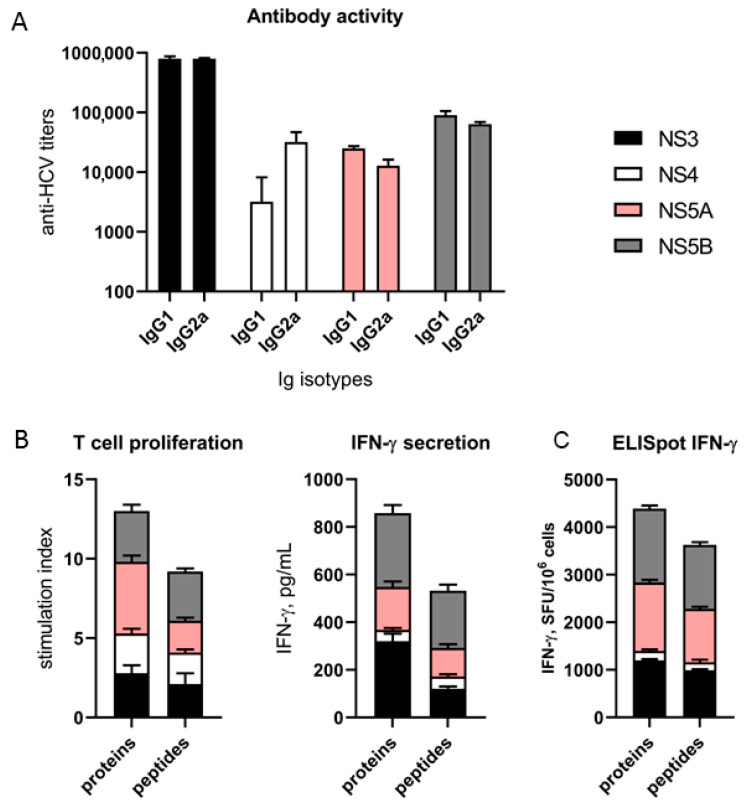
The candidate vaccine induces an effective immune response in DBA/2J mice. HCV recombinant proteins, plasmid, and a complex adjuvant were administered simultaneously subcutaneously at the root of the tail three times at 2-week intervals. (**A**)—humoral response, the levels of anti-HCV antibodies in the sera of mice are expressed as endpoint titers in ELISA; data presented as geometric mean titers of IgG1 and IgG2a antibody ± standard deviations (SD). (**B**)—results of the reactions of the lymphocytes of immunized mice to stimulation with recombinant proteins and peptides in vitro: proliferative activity is expressed as stimulation indexes (SI); secretion of IFN-γ in ELISA—in pg/mL; (**C**) number of IFN-γ-synthesizing cells in the ELISpot reaction—in the number of spots per 10^6^ cells. Data are presented as means ± SD.

The recombinant HCV proteins and the plasmid were co-administered together with the complex adjuvant subcutaneously at the root of the tail using a three-dose regimen at two-week intervals. Analysis of the humoral response in three independent experiments (Figure 4A) demonstrated that the candidate vaccine elicited a robust humoral response in DBA/2J mice, characterized by high titers of both IgG1 and IgG2a isotypes. While IgG1 titers were comparable to those observed in C57BL/6 mice, IgG2a levels in the DBA/2J strain were markedly higher, exceeding C57BL/6 titers by more than three orders of magnitude (compared with Figure 2; *p* < 0.05, Mann–Whitney test).

Evaluation of cellular immunity (Figure 4B) revealed that antigen-specific lymphocyte proliferation following restimulation with the recombinant proteins and peptides was lower in DBA/2J mice compared to C57BL/6 mice (compared with Figure 3A). However, the functional activity of lymphocytes, such as their ability to synthesize and secrete IFN-γ, remained high and was comparable to that of C57BL/6 mice (compared with Figure 3B,C).

Thus, the developed combination of HCV antigens and the adjuvant induces effective humoral and cellular responses to B- and T-cell epitopes of non-structural HCV proteins in mice of different inbred lines, with the immune response being more polarized towards Th2 in DBA/2J mice compared to C57BL/6 mice.

### 3.4. Analysis of Cell Populations in the Spleens of Immunized Animals

The final step was to compare the relative content of cell populations in the spleens of C57BL/6 mice immunized with different constructs from the HCV composition. Animals were immunized subcutaneously (three doses) with the complex adjuvant (PM + PG) in combination with recombinant proteins NS3, NS5A, NS5B (group 1), plasmid pcNS3-NS5B (group 2), recombinant proteins and plasmid simultaneously (group 3), and saline (group 4, control). All groups contained 8 mice in each of three independent experiments. The analysis was performed using flow cytometry with fluorochrome-labeled antibodies to clusters of differentiation.

Table 2 shows that no differences were found in the relative number of CD8^+^ and CD4^+^ lymphocytes. However, significant changes were registered within the CD4^+^ subpopulations. Subpopulations were characterized in accordance with the current guidelines [40] that define CD4^+^/CD62L^high^/CD44^high^ cells as central memory T-cells, CD4^+^/CD62L^low^/CD44^high^ as effector T-cells, and CD4^+^/CD25^+^/FoxP3^+^ as regulatory T-cells (Tregs). However, here Treg levels were assessed as CD4^+^/CD25^+^, as our previous study demonstrated that approximately 80% of activated CD4^+^CD25^+^ T cells are the *bona fide* regulatory T cells (Tregs) (CD4^+^CD25^+^FoxP3^+^) [35].

**Figure 5:**
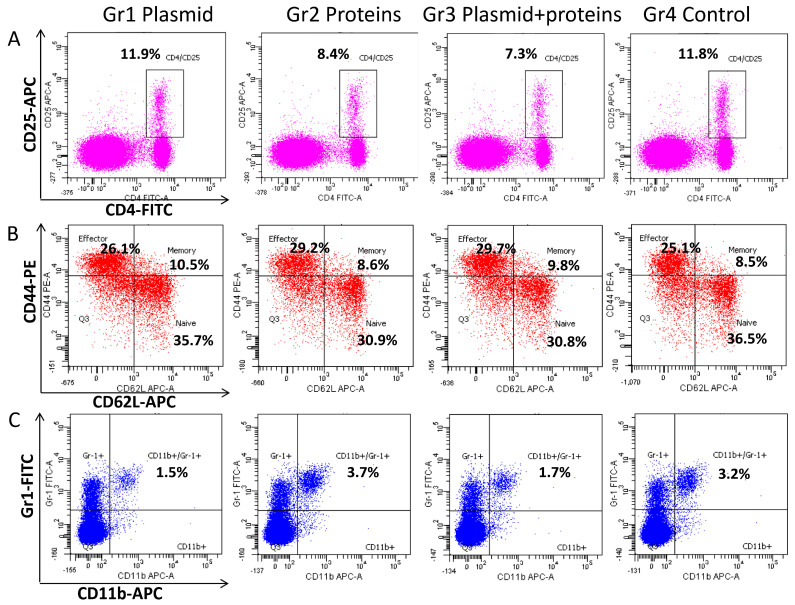
A comparative analysis of the proportion of cells in the spleens of immunized mice. Splenocytes from the immunized animals were analyzed with anti-CD4 and anti-CD25 antibodies (**A**) using Mouse Naïve/Memory T Cell Panel kit (**B**) or with anti-CD11c, anti-CD11b, and anti-Gr1 antibodies (**C**) by multicolor flow cytometry. Representative dot plots and the number of CD4/CD25-positive cells are expressed in percentages (**A**); representative dot plots and values of the number of CD4^+^ naïve (CD62 ^high^/CD44 ^low^), effector (CD62 ^low^/CD44 ^high^), and memory (CD62 ^high^/CD44 ^high^) T cells subsets are expressed in percentages (**B**); typical dot plots and values of the number of MDSC expressing CD11b and Gr1, and negative for the marker of DC (CD11c) are expressed in percentages (**C**). Representative dot plots of the number of designated cells are shown as percentages.

Therefore, in mice receiving either the proteins (group 2) or the combined DNA-protein vaccine (group 3), there was a statistically significant reduction in levels of activated CD4^+^/CD25^+^ cells that represent the phenotype of regulatory T cells (Tregs) compared to the control group (Table 3, Figure 4A). Together with the abovementioned data, this allows us to assume that the vaccine reduces the proportion of Tregs. However, we should admit that a more precise measurement of Tregs will be needed during preclinical studies. Furthermore, in the spleens of mice in groups 1 and 3, there was a statistically significant increase in the relative number of memory T cells (Table 2, Figure 5B). The proportion of effector T cells was higher in group 2, while the proportion of naive T cells was lower in group 3. The relative content of myeloid cells such as dendritic cells (DC), granulocytes, and macrophages was similar in all groups. Importantly, the proportions of myeloid-derived suppressor cells (MDSCs) were significantly reduced in groups 1 and 3 (Table 2, Figure 5C).

To sum up, the combination of HCV immunogens with a complex adjuvant PM + PG increases the proportion of CD4^+^ memory T cells and reduces the proportions of suppressor cells, such as Tregs and MDSCs. When HCV recombinant proteins and plasmid were administered separately, only a few of the described changes were observed in these cell populations.

## 4. Discussion

Hepatitis C virus (HCV) exhibits primary tropism for hepatocytes, though replication has also been documented in extrahepatic tissues [41,42,43,44]. The HCV replication machinery comprises a multisubunit complex tethered to the endoplasmic reticulum (ER) membrane [45,46,47]. It is composed of a few copies of antigenomic viral RNA, multiple genomic RNA molecules, and several hundred copies of each non-structural (NS) protein [47,48]. The NS4B protein induces the reorganization of ER membranes into “membranous webs” on which it serves as a scaffold for the replicase. Other essential components include NS3 (serine protease and helicase/NTPase) and its cofactor NS4A, the regulatory NS5A protein, and NS5B, which is an RNA-dependent RNA polymerase [49,50]. Modern pangenotypic direct-acting antivirals (DAAs) specifically block the functions of NS3, NS5A, and NS5B [51,52]. Their diverse immunogenic profiles and indispensable roles in the HCV life cycle suggest that they can be combined in a subunit vaccine. A high HCV-specific cellular response to CD4^+^ and CD8^+^ T-cell epitopes of non-structural proteins can limit infection, eliminate HCV-infected cells, and prevent the transition of an active infection to chronicity [14,17,18,53]. Therefore, these proteins and their coding sequences have been used for vaccine design by various groups. In contrast, NS4 was shown to stimulate CD4^+^ and CD8^+^ T cells less significantly than the other non-structural proteins [54]. In addition, NS4A and NS4B may inhibit host protein synthesis during translation [55]. Accordingly, NS3, NS5A, and NS5B were administered as both recombinant proteins and DNA; the NS4 sequence was exclusively presented via the pcNS3-NS5B plasmid.

The identification of an optimal platform for an HCV vaccine remains an active area of investigation. While recombinant protein-based subunit vaccines are widely used, their inherent low immunogenicity necessitates the use of potent adjuvants to modulate the immune response. Conventional adjuvants, such as aluminum salts [56,57] or water-in-oil emulsions such as MF59 and Montanide [56,57,58,59,60,61] typically polarize the response toward a Th2 phenotype, favoring humoral immunity [22]. Another group comprises adjuvants that directly affect immune cells by targeting pattern recognition receptors (PRRs) belonging to the TLR and NLR families. They can induce a Th1-type response, as shown for CpG-containing compounds that affect TLR9 on plasmocytoid DC and B lymphocytes. CpG has been shown to enhance lymphocyte proliferation, DC activation, and cytokine and immunoglobulin secretion [62]. Our findings demonstrate for the first time that a complex adjuvant system leveraging the synergistic activation of TLR4 and NOD1/NOD2 receptors significantly outperforms the licensed CpG adjuvant across multiple immunological parameters. This high immunostimulatory activity, previously observed for individual proteins [28], was confirmed here using a combination of NS3, NS5A, and NS5B proteins.

DNA vaccines stimulate primarily the cellular immune response (mainly CD8^+^) and are non-toxic. However, achieving robust transgene expression often requires optimization of the plasmid architecture, addition of immunostimulating molecules, and usage of advanced delivery methods such as electroporation [63]. Comparative studies have shown the advantages of combining two recombinant products (proteins and DNA) in a vaccine [59,64], but the issue of immunization schemes has not been resolved. Here we show that the most effective immunization regimen is a triple subcutaneous injection of recombinant proteins and a DNA vaccine simultaneously mixed with a complex adjuvant. It should be noted that the cellular response to individual constructs was also higher with subcutaneous administration compared with intramuscular administration, which was especially evident when analyzing the T-cell response of CD8^+^ lymphocytes to peptides. For DNA vaccines, it has been shown that the method of administration directly affects the direction of the immune response. This suggests that intradermal and subcutaneous injection of DNA attracts more antigen-presenting cells and induces a multifunctional cellular immune response [63].

The new vaccine composition induced a significantly more intense immune response compared to that we had previously proposed with similar specific HCV components (NS3, NS5B proteins, and the same DNA construct) [27]. The main difference concerns adjuvants and methods of injection: in the comparison study, the plasmid was injected intramuscularly in a mixture with the pcGM-CSF gene as an adjuvant, and recombinant proteins—subcutaneously in combination with IFN-α or GMDP. Table 3 demonstrates that the new regimen induced substantially higher antibody titers, with a 90-fold increase in response to NS5B (IgG1) and NS4 (IgG2a). However, it should be noted that these antibodies should have no neutralizing activity, as virions lack non-structural viral proteins. Furthermore, IFN-γ production and the levels of IFN-γ-secreting cells in response to stimulation of lymphocytes with NS3 and NS5B proteins significantly exceeded these values in the group of mice immunized with the new vaccine composition compared to the “old” regimen. The average increase in the immune response exceeded 30-fold (excluding the response to NS5A, as this protein was not administered to the comparison group of mice). Importantly, the developed vaccine composition induced a balanced Th1/Th2 response.

The combined effect of two HCV products of different nature can be synergistic, additive, or antagonistic. Our experimental data showed that in group 6, the vaccine components acted synergistically or additively; in group 7, both additively and antagonistically. A detailed study of the causes of such effects requires special additional experiments. Interesting results were obtained earlier in the study of the HIV vaccine. The authors compared the immunogenicity and protective efficacy of an HIV vaccine comprising env and gag DNA and Env proteins by co-administration of the vaccine components in the same muscles or by separate administration of DNA + protein in contralateral sites in rhesus macaques. Only macaques in the co-administration vaccine group were protected. The authors suggest that simultaneous recognition, processing, and presentation of DNA + protein in the same draining lymph nodes play a critical role in the development of protective immunity [65].

We hypothesized that the effectiveness of the immune response to the combination of HCV recombinant proteins and DNA may be related to quantitative and qualitative changes in immune cell populations. Indeed, the combination of HCV immunogens with a complex adjuvant increased the proportion of CD4^+^ memory T cells (i.e., CD4^+^/CD62L^high^/CD44^high^). These data allow us to assume that the vaccine composition stimulates the formation of CD4^+^ memory T cells. Moreover, it significantly reduced the relative content of Tregs and MDSC populations. It should be noted that separate administration of proteins and plasmid led to a reduction of only one population: Tregs or MDSCs (Table 2). MDSCs are a heterogeneous population of immature myeloid cells with a powerful suppressive potential. In patients with HCV, an increase in MDSC proportions has been described; these cells suppress the proliferation of CD4^+^ and CD8^+^ lymphocytes, NK cells, and the production of IFN-γ [66]. Tregs also exhibit an immunomodulatory effect. In patients with chronic hepatitis C, there is a significant increase in the differentiation of CD4^+^ into Tregs bearing CTLA-4 and FoxP3 markers. Tregs produce the anti-inflammatory cytokine IL-10, which inhibits the development of a Th1-type immune response. The activation of Tregs was assumed to prevent the elimination of HCV, to increase viral load, and to contribute to the chronicity of the infection [67,68]. Li J et al. (2024) reported that the gene expression profiles of CD8 T cells after DAA remained similar to those before DAA, indicating persistent disruption of gene expression for a long time after virus removal [69]. The use of antiviral vaccines capable of normalizing Tregs activity is one of the promising directions in the fight against hepatitis C: this dual action is especially useful in chronic infections, when immune modulation is crucial [70]. The effective adaptive immune response observed appears to be the result of both stimulation of effector cells and reduction in excessive activity of suppressor cells. Previously, we showed that inhibition of Tregs in vitro leads to a significant increase in IFN-γ secretion by immunized mice in response to HCV antigens [24]. The results indicate that the choice of vaccine components and vaccination regimens plays a crucial role in determining the strength, breadth, and direction of immune responses against HCV, and successful combinations can optimize the immune response to multiple targets.

Thus, the developed vaccine composition exhibits a pronounced immune response. Therefore, the next step would be to verify the neutralization of produced antibodies in cell culture (HCV) [71] or animal models [72]. Unfortunately, these animal models are of limited availability in many countries. In addition, these animals are non-immunocompetent and may have only a partially humanized immune system. This would pose another caveat for the evaluation of anti-HCV immune response. Once the vaccine combination is shown to protect animals against the virus, it will open the path to full preclinical and then clinical studies.

## 5. Conclusions

1. The triple subcutaneous immunization of animals with a mixture of pcNS3-NS5B DNA, recombinant NS3, NS5A, NS5B proteins, and a new complex adjuvant is an effective vaccine composition for inducing humoral and cellular responses in mice of different genetic lines.
2. A complex adjuvant, a combination of Polymuramyl^®^ and Pyrogenalum^®^ (NOD1/NOD2 and TLR–4 agonists, respectively), stimulated the immune response to three non-structural HCV proteins (NS3, NS5A and NS5B) significantly more effectively than the licensed CpG ODN adjuvant (TLR-9 agonist).
3. The developed vaccine composition increases the proportion of CD4^+^ memory T cells and reduces the proportions of suppressor cells—Tregs and MDSCs.

## Figures and Tables

**Table 1:** Schemes for introducing the pcNS3-NS5B plasmid and recombinant NS3, NS5A, and NS5B proteins.

Mouse Group	pcNS3-NS5B Plasmid	Recombinant Proteins	PM + PG Adjuvant
1 (*n* = 5)	IM	no	IM
2 (*n* = 5)	SC	no	SC
3 (*n* = 5)	no	IM	IM
4 (*n* = 5)	no	SC	SC
5 (*n* = 6)	IM	IM	IM
6 (*n* = 6)	SC	SC	SC
7 (*n* = 6)	IM	SC	IM+ SC
8 (*n* = 4)	no	no	IM+ SC
9 (*n* = 5)	no	no	no

C57BL/6 mice were immunized three times at 2-week intervals with either individual vaccine components (recombinant NS3, NS5A, and NS5B HCV proteins at a dose of 2 μg/mouse each or pcNS3-NS5B plasmid at a dose of 100 μg/mouse) or a combination of these components. All components were mixed with a complex adjuvant PM + PG at doses of 20 μg/mouse and 15 μg/mouse, respectively. The preparations were administered intramuscularly (IM) and/or subcutaneously (SC).

**Table 2:** A comparative analysis of the proportion of cells in the spleens of immunized mice.

Cell Population	Marker	Group 1. Plasmid	Group 2. Proteins	Group 3. Plasmid + Proteins	Group 4. Control (Saline)
Th	CD4^+^	24.2 ± 1.7	20.2 ± 2.2	21.5 ± 1.7	21.7 ± 2.9
CTL	CD8^+^	14.7 ± 1.5	13.1 ± 2.2	12.9 ± 0.7	13.8 ± 1.8
Activated T cells	CD4^+^/CD25^+^	10.4 ± 1.9	8.2 ± 1.2 *	7.9 ± 0.8 *	10.6 ± 0.9
Memory CD4^+^ T cell	CD4^+^/CD62L^high^/CD44^high^	9.4 ± 0.7 *	8.8 ± 1.0	9.2 ± 0.4 *	8.2 ± 0.3
Effector CD4^+^ T cell	CD4^+^/CD62L^low^/CD44^high^	23.9 ± 2.2	28.1 ± 1.7 *	25.6 ± 1.1	24.7 ± 0.9
Naïve CD4^+^ T cell	CD4^+^/CD62L^high^/CD44^low^	36.3 ± 1.9	32.7 ± 4.0	30.6 ± 0.4 *	35.2 ± 1.4
Granulocytes and macrophages	CD11c(-)/Gr1^+^	4.2 ± 1.0	4.5 ± 0.5	3.9 ± 0.7	4.1 ± 0.6
Dendritic cells	CD11c^+^	2.0 ± 0.1	1.8 ± 0.2	2.0 ± 0.4	2.1 ± 0.2
MDSC	CD11c(-)/CD11b^+^/Gr1^+^	1.5 ± 0.4 *	3.4 ± 0.6	1.9 ± 0.2 *	3.0 ± 0.2

Splenocytes from the immunized mice were stained with fluorochrome-labeled antibodies to CD4, CD25, CD8, CD11c, CD11b, Gr1, and with the Mouse Naïve/Memory T Cell Panel kit, and analyzed by multicolor flow cytometry. To determine MDSC, a population of lymphocytes was isolated, and cells from this gate were used to identify CD11c(-) cells. Dot plot CD11c(-) was used to determine the proportion of Gr-1+, CD11b+ cells. Values that statistically significantly (* *p* < 0.05, Student’s *t*-test) differ from the control group 4 are highlighted.

**Table 3:** Comparative analysis of the immune response to the administration of HCV recombinant proteins and pcNS3-NS5B plasmid to DBA/2J mice using different adjuvants and different immunization methods.

Assay	Response to HCV Proteins	Plasmid + Proteins with PM + PG (SC)	Plasmid with pcGM-CSF (IM), Proteins with IFN-α (SC) [27]	The Multiplicity of Differences (Folds)
ELISA: the titer of antibodies of the IgG1 isotype *	NS3	790,035 ± 71,000	32,900 ± 3300	24
	NS4	3200 ± 5000	98 ± 76	333
	NS5A	25,080 ± 2500	118 ± 65	213
	NS5B	90,050 ± 15,700	963 ± 56	94
ELISA: the titer of antibodies of the IgG2a isotype *	NS3	810,020 ± 23,040	80,070 ± 12,000	10
	NS4	32,000 ± 15,000	285 ± 28	112
	NS5A	32,030 ± 15,010	158 ± 106	203
	NS5B	64,020 ± 5400	7562 ± 2128	8.5
ELISA: IFN-γ secretion (pg/mL) **	NS3	320 ± 32	9 ± 3	36
	NS4	48 ± 8	29 ± 12	1.7
	NS5A	180 ± 32	11 ± 6	16
	NS5B	310 ± 34	12 ± 4	26
ELISpot: the number of IFN-γ-synthesizing cells (spots/10^6^ cells) **	NS3	1196 ± 30	135 ± 23	8.9
	NS4	200 ± 36	106 ± 31	1.9
	NS5A	1440 ± 59	28 ± 15	51
	NS5B	1552 ± 70	35 ± 18	44

DBA/2J mice were immunized three times at 2-week intervals with HCV recombinant proteins and pcNS3-NS5B plasmid in a mixture with specified adjuvants. The preparations were administered intramuscularly (IM) or subcutaneously (SC); data presented as: * geometric mean ± SD and ** means ± SD.

## Data Availability

The original contributions presented in this study are included in the article. Further inquiries can be directed to the corresponding author(s).
